# Closed Genome Sequence of Yavru, a Novel Arthrobacter globiformis Phage

**DOI:** 10.1128/MRA.00986-21

**Published:** 2021-11-11

**Authors:** Meliha Ulker, Fardeen A. Siddiqui, Thomas J. Gerton, Rachel E. Anastasi, Dylan J. Conroy, Ethan G. Edwards, Isabelle E. Laizure, Joshua D. Reynolds, Kelsie Duggan, Kristen C. Johnson, Kyle S. MacLea

**Affiliations:** a Biotechnology Program, University of New Hampshire, Manchester, New Hampshire, USA; b Graduate Program in Biotechnology: Industrial and Biomedical Sciences, University of New Hampshire, Manchester, New Hampshire, USA; c Biology Program, University of New Hampshire, Manchester, New Hampshire, USA; d Department of Life Sciences, University of New Hampshire, Manchester, New Hampshire, USA; Queens College CUNY

## Abstract

We characterized the complete genome sequence of actinobacteriophage Yavru (*Siphoviridae*), a cluster FE bacteriophage infecting Arthrobacter globiformis NRRL B-2979; it was 89.5% identical to cluster FE phage Whytu, with a capsid width of 50 nm and a tail length of 90 nm. The genome was 15,193 bp in length, with 23 predicted protein-coding genes.

## ANNOUNCEMENT

**FIG 1:**
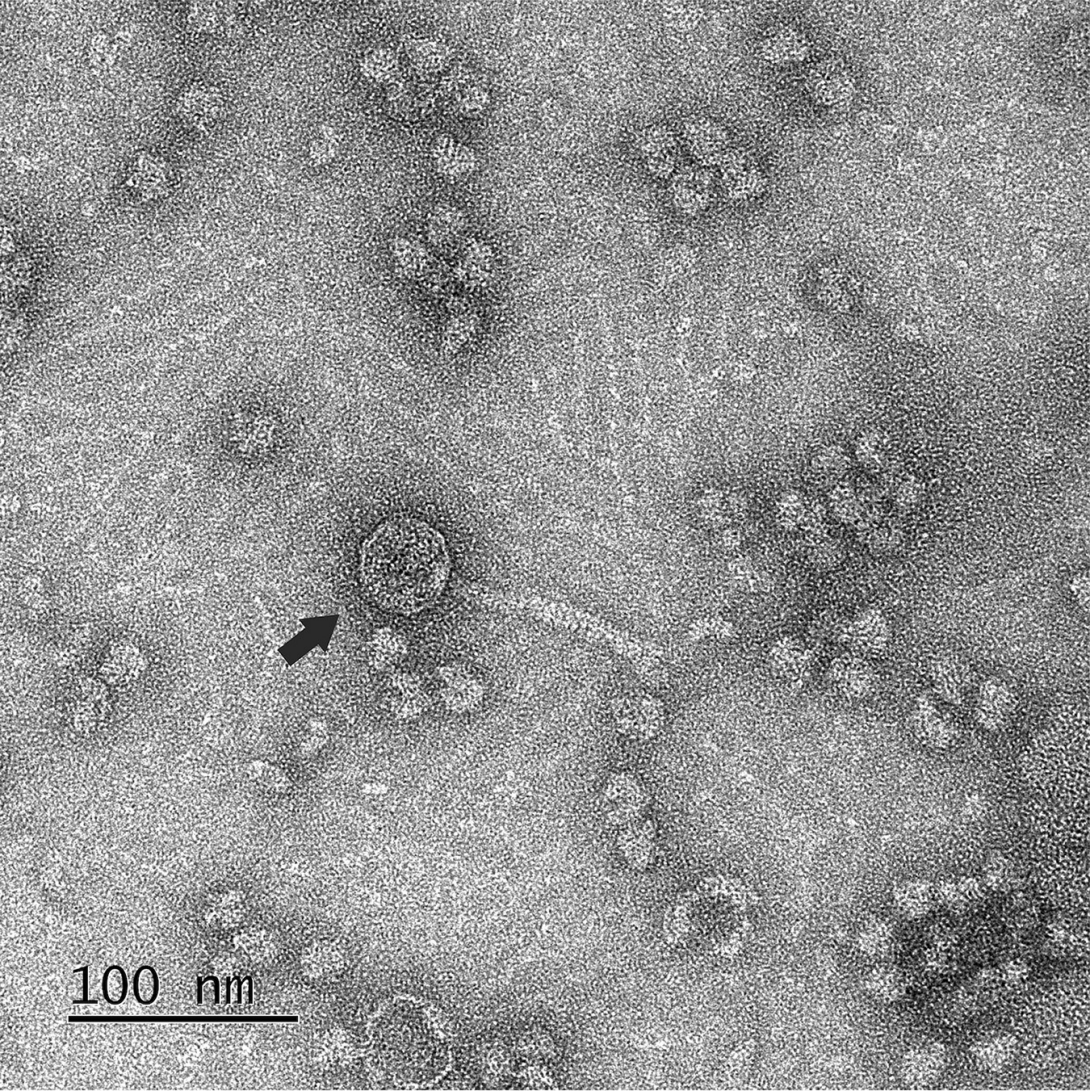
Transmission electron micrograph of Arthrobacter globiformis bacteriophage Yavru. A high-titer phage lysate was negatively stained with 1% uranyl acetate on a carbon conductive PELCO tab and imaged with a Tecnai F20 microscope at the Dartmouth Electron Microscope Facility. A siphoviral phage particle (capsid head, attached to a long noncontractile tail) is indicated by a black arrow. Scale bar = 100 nm.

Yavru is a lytic actinobacteriophage infecting the host strain Arthrobacter globiformis NRRL B-2979; it was isolated from grainy garden soil in Bedford, New Hampshire, USA (42.958N, 71.5642W), in September 2019. Isolation, purification, and amplification of Yavru were performed using the Science Education Alliance-Phage Hunters Advancing Genomics and Evolutionary Science (SEA-PHAGES) direct isolation protocols (1). Briefly, on double-layer peptone-yeast-calcium agar (PYCa) with 1 mM calcium chloride, 10 μg/ml cycloheximide, and 40% dextrose, Yavru produced 1-mm circular plaques (clear in the middle, with surrounding turbidity) on lawns of A. globiformis grown at 26°C in normal room atmosphere. A high-titer lysate of Yavru (5 × 10^12^ PFU/ml) was deposited on PELCO carbon conductive tabs (16084-1; Ted Pella, Redding, CA) and negatively stained with 1% uranyl acetate as described previously (2, 3). Under transmission electron microscopy (TEM) with a Tecnai F20 microscope (Dartmouth Electron Microscope Facility, Hanover, NH, USA), the capsid head of Yavru measured 50 nm in diameter, and its siphoviral noncontractile tail measured 90 nm (Fig. 1).

Yavru DNA was extracted from a high-titer lysate using the Wizard DNA clean-up kit (A7280; Promega, Madison, WI, USA). A sequencing library was produced using the Ultra II FS kit (E7805S; New England Biolabs, Ipswich, MA) with dual-indexed barcoding. Yavru yielded 540.8 thousand single-end 150-base reads by Illumina MiSeq sequencing (Pittsburgh Bacteriophage Institute, Pittsburgh, PA, USA), and Newbler v2.9 assembled 100,000 randomly chosen reads into a single contig, resulting in 5,083-fold coverage.

The genome of Yavru was annotated (4) using DNA Master v5.23.3 (http://cobamide2.bio.pitt.edu/computer.htm) (5), with predicted coding sequence analysis by GLIMMER v3.02 (6), GeneMarkS v2.0 (7), Phamerator (8), and Starterator v1.0.1 (https://github.com/SEA-PHAGES/starterator). Twenty-three protein-coding genes were found in Yavru using BLAST (9), HHpred (10), and conserved synteny. No tRNA or transfer-messenger RNA genes were located by ARAGORN v1.2.38 (11) or tRNAscan-SE v2.0 (12). All software was used with default settings.

Yavru has a 15,193-bp genome size and currently has the sixth smallest phage genome of the 1,019 known *Arthrobacter* phages at PhagesDB (13). All of Yavru’s 23 genes were transcribed in a forward direction, and 16 (70%) have assigned functions. Yavru has classic siphoviral genes, including portal protein, terminase, and head-to-tail proteins (14), and lytic genes like endolysin and holin (15). Because it also lacks tail tube, sheath, and lysogeny genes, its classification as a lytic siphoviral bacteriophage, like other close relatives (16), is clear. Yavru is one of 7 known phages in cluster FE, sharing the highest average nucleotide identity (ANI) (as computed by the ANI Calculator [17]) (89.5%) with phage Whytu, which was isolated from Marina del Rey, California, USA, more than 2,500 miles from Yavru’s isolation site.

Horizontal gene transfer (HGT) can be inferred by observing GC content differences between a virus and its host (18), with bacteriophages customarily showing equivalent or reduced GC content (19, 20). Deviations from this standard can be employed as potential evidence for HGT. Yavru has an average GC content of 64.3%, and Arthrobacter globiformis NRRL B-2979 has an average GC content of 66.2% (similar to those of published A. globiformis strains [21]). Gene 16 of Yavru, a MerR-like helix-turn-helix DNA binding domain protein, possesses a significantly higher GC content (73.1%) and therefore is a possible horizontal acquisition from a foreign genome.

### Data availability.

The genome sequence of the bacteriophage Yavru (https://phagesdb.org/phages/Yavru) has been deposited in DDBJ/ENA/GenBank under accession number MT889364. The raw Illumina data from BioSample SAMN18929603 were submitted to the NCBI Sequence Read Archive (SRA) under SRA accession number SRX10779622.
